# Is the core-periphery labour market structure related to perceived health? findings of the Northern Swedish Cohort

**DOI:** 10.1186/1471-2458-11-956

**Published:** 2011-12-27

**Authors:** Anna-Karin Waenerlund, Per E Gustafsson, Pekka Virtanen, Anne Hammarström

**Affiliations:** 1 Department of Public Health and Clinical Medicine, Family Medicine, Umeå University, SE-901 85 Umeå, Sweden; 2 School of Health Sciences, University of Tampere, Tampere, Finland; 3 Department of Public Health and Clinical Medicine, Epidemiology and Global Health, Umeå University, Umeå, Sweden

**Keywords:** Cohort, Employment, Health, Psychological distress, Public health

## Abstract

**Background:**

There is controversy as to whether peripheral employment is related to poor health status or not. This study aims at examining whether 1) the accumulation of time in peripheral labour market positions is associated with psychological distress and poor or average self-rated health; 2) the proposed association is different among women than among men.

**Method:**

Participants in the 1995 and 2007 follow-up surveys of the Northern Swedish Cohort (n = 985) completed self-administered questionnaires about psychological and general health and about employment positions during the follow-up years. Associations between 12 year peripheral labour market positions (no, low, medium and high exposure) and health were examined using logistic regression.

**Results:**

Exposure to peripheral employment was positively related to psychological distress in both women and men (*p*-values for trend < 0.001). Adjustment for sociodemographics and psychological distress at baseline, as well as for unemployment and being out of the labour market at the follow-up, resulted in attenuation of the odds ratios, particularly in the group with high exposure to peripheral employment, although results remained significant in men in the fully adjusted model. Women and men with high exposure to peripheral employment had high odds of poor or average self-rated health, but the association was rendered non-significant after adjustment for the covariates.

**Conclusions:**

Our findings suggest that exposure to peripheral employment positions has an impact particularly on mental health, partly due to the over-representation of other unfavourable social and employment conditions among those with substantial exposure to peripheral employment.

## Background

Intensified competition in the global market requires organisations to improve their profitability. One way to cut costs is to increase numerical flexibility [1]: organisations tend to reduce the proportion of permanent employees and prefer temporary contracts [2]. At the European level, 14% of the employees had a temporary contract in 2009 [3], and the European Commission emphasises the importance of labour market flexibility in Europe [4]. This development gives reason to regard temporary employment as an important topic of public health research [5], in particular as uncertainty prevails about the health consequences of temporary employment in the flexible labour market. There is evidence suggesting that, compared to permanent employees, temporary employees might suffer from poorer general and mental health [6-8]. However, other studies have shown no contract-related differences e.g. in psychological health [9,10] or in self-rated health [10,11].

In today's flexible post-industrial labour market a simple dichotomy of employed and unemployed is long gone, as it is common to move between different occupations and careers with different types of employment contracts. Notions of temporary employment as an exposed employment condition somewhere in between permanent employment and unemployment partly address these issues, but fail to take the substantial heterogeneity among temporary contracts into consideration. To address the complexities of the current labour market, Aronsson [12] has developed a conceptual model in which employment contracts are differentiated based on degree of job insecurity along an axis between the core and the periphery of the labour market. The core-periphery model [8,13,14] aims to capture this heterogeneity by regarding the permanent employees as the 'core work force' with favourable working conditions and temporary employees as the 'periphery work force' with reduced benefits. The structure of the model is based on aspects along the core-periphery axis, ranging from the core of permanent workers, to employment on projects/probationary employment, substitutes, and last the seasonal and on-call workers being closest to the periphery. The model suggests that the core represents the group with most secure conditions, with insecurity increasing the further from the core and the closer to the periphery one comes. In addition, the core-periphery axis also describes increasingly unfavourable working conditions the further from the core one comes, e.g. in terms of duration of contract, skill development, influence on decision-making, education, and support from supervisors [15]. Project and object employment resembles the working conditions in the core, substitute contracts represents somewhere in the middle and on-call and seasonal workers are the groups with working conditions that least resemble the working conditions of the core [12]. The model also suggests that there is a health gradient running along the core-periphery axis, with worse health among those closer to the periphery. In the present study we consider the relative hierarchy between different employment contracts along the core-periphery axis, accumulated across a 12 year period, as a determinant for health.

Along with the increase of unfavourable conditions towards the periphery, it would be expected that there are corresponding gradients in the health of the employees [13]. However, there is lack of studies that take into account how far in the periphery, or how adverse, the position is. Moreover, most studies have utilised cross-sectional designs, although the duration in peripheral employment might be significant for health impacts to develop [14].

In studies of employment it is very important to consider potential effects of previous health as there is a risk of selection, e.g. individuals with good health stay in or move towards more core jobs, whilst those with poorer health are at greater risk of moving towards the periphery of the labour market [10]. Those with peripheral employment might thus be particularly vulnerable to health selection, resulting in a career trajectory further towards the periphery [10]. As many previous studies on the health impact of temporary employment have applied a cross-sectional design, health selection cannot be ruled out [6]. Gender discrimination on the labour market has led to women being compelled to take on less beneficial non-standard employments [15], with temporary employment being more common among women than men in Europe, North America as well as Asia [15,16]. However, so far research has shown some inconsistencies as to whether women's and men's health are affected by temporary employment to a similar or different degree [6,16], As such, whether temporary employment, particularly over time, affects women's and men's health to the same degree is a question that needs to be addressed.

Previous research in Sweden within the field suggests that women are over-represented in at least some peripheral employment contracts [13]. Only a few studies have done analyses stratified by gender [16,17], and public health researchers have emphasised that the issue of gender in relation to type of employment and health has been overlooked [15].

The present longitudinal study concerns these so far unexplored questions of the health impacts of temporary employment. We have developed Aronsson's core-periphery model by adding the dimension of time as well as including self-employed, active in labour market programme and other temporary employment contracts. Based on measurement of the stay in different positions in the core-periphery labour market structure during a 12 year period, this study aims at examining whether 1) the accumulation of time in peripheral labour market positions is associated with psychological distress and poor or average self-rated health; 2) the proposed association is different among women than among men.

## Methods

### Population

The study was initiated in 1981 and included all pupils in their last year of compulsory school (n = 1083), in the medium-sized industrial town of Luleå in northern Sweden (95% of the cohort were born in 1965). Since 1981 four additional follow-ups have taken place. The sample has been shown to be fairly representative of Sweden as a whole with respect to sociodemographic factors, socio-economic factors and health status [18]. The attrition rate in the most recent follow-up from 2007 was low; 94% (n = 1005) of the original cohort who were still alive (n = 1071) participated. The analyses in this study were based on all participants who were ever employed between 1996 and 2007 (n = 985, 473 women and 512 men).

### Procedures

Comprehensive questionnaires were used as the main assessment method, completed by the participants in 1995 and 2007 with similar procedures at both surveys. All participants were invited to school reunions at their old school. The participants were given information about the study and completed the self-administered questionnaires. The questionnaires were mailed to the participants who did not attend the school reunion. In case of non-response and for those who preferred to answer the questionnaires by phone, supplementary telephone interviews were conducted. The study was approved by the Regional Ethical Review Board in Umeå [no. 07-057].

### Measurements

#### Exposure

##### Peripheral Employment Score

The peripheral employment score was measured by the self-administered questionnaire as completed by participants in 2007, by applying an extended version of Aronsson's original core-periphery model [12]. This extended version of Aronsson's model also includes self-employed, active in labour market programme and other temporary employment contracts and also incorporates the dimension of time. Although being in a labour market programme is not considered employment per se, it might share some important aspects with peripheral employment more than with unemployment (e.g., regarding latent functions of employment), and was therefore included in the exposure. Participants' labour market position from 1996 to 2007 was measured with a matrix consisting of columns representing half-year periods and lines representing different labour market positions. With the instruction 'During which periods have you been employed permanently or have had some type of temporary job contract or have been out of job?' the respondents were advised to choose between 11 employment options for each half-year period: 'permanently employed', 'entrepreneur', 'employed in project', 'substitute', 'probationary employment', 'on-demand worker', 'seasonal worker', 'temporary employee for other reasons', 'active in labour market programme, 'unemployed', 'out of the labour market'. The order of the different employment contracts is based on the relative hierarchy in relation to degree of insecurity, reaching from the most core to the most peripheral employment contract.

Each response option was given a score for each half-year period according to the core-periphery structure: permanent (coded as 0), self-employed (= 1), project/object employed (= 2), probationary (trial period of maximum 6 months after which employer decides whether the employee is hired permanently or let go) (= 3), substitute (temporary replacement of ordinary employee, e.g. filling in while an ordinary employee is on parental leave) (= 4), seasonal (= 5), on-call (to meet emergency requirements) (= 6), active in labour market programme (= 7) and other temporary employment (= 4; the mean score among temporary employment contracts). If more than one option per column was marked, the alternative closest to 0 (i.e., closest to the core) was used.

In order to yield a cumulative measure of cumulative peripheral employment reflecting the entire 12 year period, the scores for all 24 half-year periods were averaged, generating a variable with range 1-7. The peripheral employment score can thus be viewed as the average degree of peripheral employment across the entire 12 year period, thus considering both duration (accumulated time in different contracts) and degree (type of temporary contract) of exposure to peripheral employment. This variable was very skewed, and in order to yield a variable more suitable for our analyses, the score was therefore divided into four groups: 0 = no experience of temporary employment; with experience of temporary employment or self-employment divided into tertiles of exposure, separately by gender: tertile 1 = 'low exposure', tertile 2 = 'medium exposure', tertile 3 = 'high exposure'. This four-level variable of cumulative peripheral employment was used in all analyses.

#### Indicators of health

*Psychological distress *at age 30 (indicator of earlier psychological distress) and 42 (outcome) was measured with a question that inquired whether the respondent during the last year had experienced symptoms of psychological distress (restlessness, concentration problems, being worried or anxious, palpitations, anxiety or panic or other nervous problems), with the response options 'yes' or 'no'. The question was derived from the 'Survey of Living Conditions' [19]. The worst quartile, reporting one or more of the six symptoms, was coded as 1 and the rest reporting none as 0 for men. For women both at age 30 and 42, the worst quartile reported 2 or more symptoms and was coded as 1 and less as was coded as 0 [20]. For use in complementary analyses, psychological distress at age 16 and 21 was used as an indicator of previous health, reporting one or more symptoms was coded as 1, no symptoms as 0 accordingly with the 75th percentile for both women and men.

*Poor or average self-rated health *at age 30 (indicator of earlier self-rated health) and 42 (outcome) was measured with one question, 'How do you rate your general health?', with response options: 'good', 'average' or 'bad' [19]. The responses were dichotomised into the quartile with the worst health (average or bad) coded as 1, and the rest (good) coded as 0 [20,21]. This question was not available at earlier ages.

#### Covariates

*Socioeconomic position *(SEP) at age 42 was based self-reported occupation, which was classified according to the Swedish socioeconomic classification (SEI) of occupational categories: [22] upper white-collar and self-employed were coded as 0, lower white-collar workers were coded as 1 and blue-collar workers were coded as 2 and were used as categorical variables in the analysis.

*Parental status *at age 42: having children was coded as 0 and no children as 1.

*Marital status *at age 42 was measured with one question: 'Are you married or do you have a live-in partner?' 'Yes' was coded as 0 and 'No' as 1.

#### Unemployment and out of labour market

*Time in unemployment *and *time out of the labour market *over the last 12 years (i.e., between age 31 and 42) were derived from the same matrix that was used to operationalise cumulative peripheral employment. Each period of unemployment and out of the labour market, representing a six-month period of time, was summarised into a continuous measure of unemployment or out of the labour market.

### Statistics

Logistic regression was used as the main statistical method. The results are presented separately for men and women. We tested whether no, low, medium or high exposure to peripheral employment was associated with poor or average self-rated health and psychological distress at age 42. Adjustments were made for the health indicators of interest at age 30, socioeconomic position, parental status, marital status and unemployment and out of the labour market.

In complementary analyses, adjustment was made for psychological distress at age 16 and 21 instead of at age 30, yielding similar but slightly more pronounced results as compared to adjusting for psychological distress at age 30 (data not shown). As results were similar and self-rated health was not collected at earlier ages, only results with adjustment for earlier health at age 30 are reported in the results section. SPSS v17 was used for all analyses.

## Results

Table 1 shows the distribution of the study variables by degree of exposure to peripheral employment. In general, experience of peripheral employment entailed a higher risk of unfavourable sociodemographic and health conditions. The higher the degree of exposure to peripheral employment, the higher was the proportion of psychological distress at age 42, in both women and men. Poor or average self-rated health at age 42 displayed a comparable but non-significant pattern in both women and men. Concerning covariates, similar general patterns were seen for psychological distress and poor or average self-rated health at age 30, with the latter also differing significantly with respect to peripheral employment except in men. There were significant differences in socioeconomic position for both genders, with the numerically highest proportion of blue-collar workers in the group with high exposure to peripheral employment. This was also the group that was most likely to be living without a partner and to have experienced longer periods of unemployment and being out of the labour market. Parental status did not differ between the groups.

**Table 1:** Distribution among women and men, % for dichotomous variables, median (iqr) for continuous variables in relation to peripheral employment score

Variable	Women				*p*-value
	**No exposure**	**Low exposure**	**Medium exposure**	**High exposure**	

	**n = 239**	**n = 81**	**n = 74**	**n = 79**	

**Dependent variables age 42**					

Poor or average self-rated health (%)	31.8	35.8	39.2	46.2	0.13

Psychological distress (%)	20.6	33.3	32.9	39.2	0.004

**Independent variables**					

Poor or average self-rated health age 30 (%)	18.4	27.5	32.9	34.6	0.007

Psychological distress age 30 (%)	13.8	25.9	33.8	31.6	< 0.001

**Sociodemographics**					0.008

Blue-collar worker (%)	28.9	23.5	29.7	48.1	

Lower white-collar worker (%)	18.8	24.7	13.5	8.9	

Upper white-collar worker (%)	52.3	51.9	56.8	43.0	

Marital status single (%)	18.5	22.2	9.5	27.8	0.031

Parental status no children (%)	14.3	9.9	9.5	8.9	0.43

Unemployment*					< 0.001

Ever unemployed* (%)	10.0	35.8	33.8	46.8	

Time in unemployment, Md (iqr)*	2.00 (1.00-3.75)	2.00 (1.00-5.00)	3.00 (1.50-5.00)	3.00 (2.00-8.50)	

Out of labour market*					< 0.001

Ever out of labour market * (%)	13.8	45.7	44.6	50.6	

Time out of labour market Md (iqr)*	4.00 (2.50-7.50)	6.00 (4.00-11.00)	5.00 (3.00-10.50)	6.00 (4.00-10.75)	

**Variable**	**Men**				***p*-value**

	**No exposure n = 313**	**Low exposure n = 65**	**Medium exposure n = 74**	**High exposure n = 60**	

**Dependent variables age 42**					

Poor or average self-rated health (%)	30.7	35.4	37.0	45.0	0.169

Psychological distress (%)	24.6	26.2	35.6	51.7	< 0.001

**Independent variables**					

Poor or average self-rated health age 30 (%)	18.1	22.2	31.5	26.7	0.061

Psychological distress age 30 (%)	20.8	27.7	36.5	36.7	0.006

**Sociodemographics**					< 0.001

Blue-collar worker (%)	40.2	28.1	23.0	56.7	

Lower white-collar worker (%)	11.3	10.9	2.7	11.7	

Upper white-collar worker (%)	48.6	60.9	74.3	31.7	

Marital status single (%)	21.8	27.7	18.9	45.0	0.001

Parental status no children (%)	21.5	24.6	16.2	35.0	0.06

Unemployment*					< 0.001

Ever unemployed* (%)	12.1	33.8	24.3	60.0	

Time in unemployment, Md (iqr)*	2.00 (1.00-5.50)	4.50 (2.00-8.25)	3.50 (2.00-5.25)	6.50 (3.00-11.25)	

Out of labour market*					< 0.001

Ever out of labour market * (%)	8.3	23.1	25.7	34.5	

Time out of labour market Md (iqr)*	5.00 (2.00-9.00)	7.00 (3.00-10.00)	7.00 (4.00-9.00)	5.00 (2.50-9.50)	

*P-values are from Kruskal-Wallis test with continuous time in unemployment/out of labour market by exposure to peripheral employment. Due to sample median = 0, descriptive statistics are reported as percentage ever unemployed/out of labour market, as well as median time in unemployment/out of labour market in the subsample with at least one spell of unemployment/being out of labour market

To examine in greater detail whether exposure to peripheral employment was cumulatively related to poor health, self-rated health and psychological distress were regressed on peripheral employment (no, low, medium and high exposure) and covariates in logistic regression models, with separate analyses for women and men (Table 2).

**Table 2:** Logistic regression analysis Odds ratios (OR) and 95% confidence intervals (CI) for psychological distress at age 42 with peripheral employment score over 12 years as main exposure (the score is divided into no exposure, low exposure, medium exposure, high exposure) after adjustment for psychological distress age 30, sociodemographic variables, unemployment and out of labour market

Peripheral employment	Women			
	**Model 0**	**Model 1**	**Model 2**	**Model 3**

No exposure	1	1	1	1

Low exposure	1.93 (1.10-3.72)	1.69 (0.92-3.11)	1.46 (0.80-2.66)	1.35 (0.71-2.57)

Medium exposure	1.89 (1.06-3.77)	1.70 (0.90-3.20)	1.55 (0.85-2.83)	1.46 (0.76-2.78)

High exposure	2.49 (1.44-4.31)	1.79 (0.98-3.29)	1.88 (1.03-3.45)	1.42 (0.73-2.74)

p-value for trend	0.001	0.034	0.033	0.228

Nagelkerke R^2^	0.041	0.194	0.071	0.209

**Peripheral employment**	**Men**			

	**Model 0**	**Model 1**	**Model 2**	**Model 3**

No exposure	1	1	1	1

Low exposure	1.09 (0.59-2.00)	1.04 (0.54-1.97)	0.87 (0.46-1.66)	0.87 (0.45-1.71)

Medium exposure	1.70 (0.98-2.92)	1.44 (0.80-2.60)	1.47 (0.84-2.56)	1.27 (0.70-2.32)

High exposure	3.28 (1.86-5.79)	2.79 (1.52-5.14)	2.26 (1.21-4.22)	2.18 (1.14-4.20)

p-value for trend	< 0.001	0.001	0.009	0.028

Nagelkerke R^2^	0.050	0.157	0.086	0.173

Model 0 crude odds ratios

Model 1 odds ratios adjusted for sociodemographic variables (socioeconomic position, marital status and parental status) and psychological distress at age 30

Model 2 odds ratios adjusted for number of periods of unemployment and out of labour market

Model 3 odds ratios adjusted for model 1+2

In unadjusted analyses with psychological distress, exposure to peripheral employment involved higher odds of psychological distress in both women and men (Table 2 Model 0). Adjusting for covariates (sociodemographics and psychological distress at age 30, Model 1) in women resulted in attenuation particularly of the OR for those with high exposure to peripheral employment. The attenuation in this specific group thus seemed to be explained by the higher risk of unfavourable conditions in the group (psychological distress at age 30, single marital status and blue-collar occupations, see Table 1). Similar adjustments in men led to an attenuation of ORs across the exposure groups, with only the high exposure group and trend across all groups remaining significant. Adjusting for duration of unemployment and out of labour market in a separate model (Model 2) resulted in attenuation of the ORs of all groups. However, the high-exposure groups and the trends were still significant in women and men, indicating that unemployment and being out of the labour market did not fully explain the observed association. Lastly, in fully adjusted models (Model 3) only the high-exposure group was significant in men, and none in women.

Results for poor or average self-rated health were notably less consistent than for psychological distress. In the unadjusted models (Table 3 Model 0), women and men with high exposure to peripheral employment had higher odds of having poor or average self-rated health; for those with low and medium exposure the risk was numerically higher but not significant. When adjusted for covariates (Models 1-3) the result did not remain significant in any of the models. [In relation to Table 2 data not shown] In model 3, psychological distress at age 30 (women OR 3.57 (2.17-5.87), men OR 3.63 (2.33-5.66)), being out of the labour market (women OR 1.07 (1.01-1.12), men OR 1.07 (1.00-1.14)) and for women marital status single (OR 3.18 (1.82-5.55)) were significantly related to psychological distress at age 42. None of the other covariates were related to psychological distress at 42 in any of the models. [In relation to Table 3 data not shown] In model 3, poor or average self-rated health at age 30 women OR 3.53 (2.22-5.61) men OR 4.06 (2.52-6.55), for women blue-collar worker (OR 1.99(1.24-3.17)) and single marital status OR (2.29 (1.33-3.95)) and for men being out of the labour market (OR 1.09 (1.02-1.17)) were significantly related to poor or average self-rated health at age 42. The only other covariate that was significant was being out of the labour market for women in model 2 (OR 1.08 (1.03-1.32)).

**Table 3:** Logistic regression analysis Odds ratios (OR) and 95% confidence intervals (CI) for poor or average self-rated health at age 42 with peripheral employment score over 12 years as main exposure (the score is divided into no exposure, low exposure, medium exposure, high exposure) after adjustment for self-rated health at age 30, sociodemographic variables and unemployment and out of labour market

Peripheral employment	Women			
	
	Model 0	Model 1	Model 2	Model 3
No exposure	1	1	1	1

Low exposure	1.20 (0.70-2.03)	1.08 (0.61-1.92)	0.94 (0.53-1.66)	0.93 (0.50-1.71)

Medium exposure	1.38 (0.81-2.37)	1.35 (0.75-2.42)	1.16 (0.66-2.04)	1.20 (0.66-2.21)

High exposure	1.84 (1.09-3.10)	1.34 (0.75-2.38)	1.45 (0.82-2.58)	1.16 (0.62-2.17)

p-value for trend	0.019	0.232	0.194	0.513

Nagelkerke R^2^	0.016	0.173	0.041	0.183

	**Men**			

	**Model 0**	**Model 1**	**Model 2**	**Model 3**

No exposure	1	1	1	1

Low exposure	1.23 (0.70-2.16)	1.20 (0.65-2.20)	1.01 (0.56-1.82)	1.07 (0.56-2.01)

Medium exposure	1.32 (0.78-2.25)	1.19 (0.66-2.14)	1.16 (0.67-2.00)	1.09 (0.60-1.98)

High exposure	1.84 (1.05-3.24)	1.41 (0.77-2.60)	1.26 (0.67-2.37)	1.26 (0.65-2.44)

p-value for trend	0.029	0.236	0.414	0.506

Nagelkerke R^2^	0.013	0.145	0.051	0.163

Model 0 crude odds ratios

Model 1 odds ratios adjusted for sociodemographic variables (socioeconomic position, marital status and parental status) and self-rated health at age 30

Model 2 odds ratios adjusted for number of periods of unemployment and out of labour market

Model 3 odds ratios adjusted for model 1+2

## Discussion

The Northern Sweden Cohort, with only six percent attrition over a 26 year period, offers a unique possibility to examine health gradients in the core-periphery structure of the labour market. In this paper we found increasingly higher odds of psychological distress among those with higher exposure to cumulative peripheral employment over a 12 year period. However, particularly sociodemographics and previous health in women and unemployment and being out of the labour market among men explained the relationships to a substantial degree, although some results were still significant in men in the fully adjusted model. The results for self-rated health were in a similar direction but numerically weaker and - except for the unadjusted analyses - non-significant throughout the models.

Most previous research has only measured exposure to peripheral employment at one point in time, and often concurrently with the outcome [14]. Cross-sectional approaches thus fail to capture the potential impact of long-term exposure to peripheral employment. Indeed, previous epidemiological research on unfavourable social conditions, e.g. economic hardship, has shown that duration of exposure might act cumulatively on health [23]. This general notion is analogous to our consideration of the duration of peripheral employment. Another key aspect that has been highlighted in research on labour market position is the relative hierarchy of employment contract types [8], such as the core-periphery structure [1,12,24]. In this study we addressed duration and degree, two aspects of the inherent heterogeneity in peripheral employment, by using a cumulative and graded approach to peripheral employment.

Earlier research reports that peripheral employment is associated with psychological distress [7,16,17], which generally corresponds to the findings in this study. We extend previous findings by demonstrating that duration and degree of peripheral employment act cumulatively on health over time. However, periods of unemployment and out of the labour market were more common among those with peripheral employment, possibly as a result of those with temporary employment being at high risk of becoming unemployed [25]. Moreover, those with peripheral employment were also more frequently single, potentially because temporary employment comprises unstable contracts which may delay partnership formation [9], and blue-collar occupations were more frequent among temporarily employed people, corresponding to some earlier research [26]. Taking these additional exposures into consideration resulted in a substantial attenuation of the association between temporary employment and distress for both women and men. Our results thus suggest that the peripherally employed also are exposed to clusters of other important risk factors for distress, and that this clustering of risk factors partially explains the worse mental health among the peripherally employed.

Previous findings on self-rated health and peripheral employment have been divergent [8,10]. Our results corroborate previous findings suggesting that there are weak associations between peripheral employment and poor self-rated health [10,17]. Although the results for self-rated health were in the same direction as for psychological distress, one might question why there would be a more consistent association for distress. Self-rated health is a broad measure, reflecting cultural, historical and biological evaluation of a person's health [27], whilst psychological distress reflects an unpleasant subjective state; a combination of physical and emotional distress such as feelings of restlessness and anxiety [28]. Previous research has shown that peripheral employment is closely linked to job insecurity and other stressors, which might more readily manifest as psychological distress [29].

Previous studies that have stratified for gender have in general found more pronounced associations between temporary or precarious employment and health in women than in men [16,17]. In contrast to these studies but in accordance with one other study [30], we found that results were quite similar in women and men, which suggests that the potential adverse health consequences of peripheral employment are not an issue solely for women. Possible explanations as to why we found similar associations among women and men, whilst some previous studies found more profound associations among women [16], could depend on how we measured the exposure, as we consider the type of contracts in greater detail than previous research [16,17,30]. For example, the divergent results could partly depend on whether women or men more frequently have the most peripheral type of contracts, with the most peripheral types of contracts having greater implications for health. As our results indicate, it might not be gender per se but type of contract that has the greatest importance for health. However, it is also possible that gender discrimination has different implications depending on the specific cultural context [16].

### Methodological considerations

Some previous studies have had difficulties obtaining a high response rate in research on temporary employees [31], which might bias the results. In studies on employment and health it is important to consider the issue of causality, e.g., if those who have been on sick-leave are more likely to end up with temporary than a permanent employment. To reduce the risk of drawing faulty conclusions regarding the direction of the relationship between temporary employment and health we have included health status at baseline in the analysis. However, it is still possible that unmeasured health selection effects could influence the results. Although there is no precise way of considering health-related selection, a common approach is to use previous health as an indicator of health-related selection [32]. It is important to note that different absolute cut-off points were used for the psychological distress outcomes in men and women. The cut-off points were based on the worst quartile vs. the rest, which implies that no direct comparison can be made between men and women regarding the absolute levels of psychological distress.

In this study the attrition rate was low, which is a major strength of the study. Previous examinations suggest that the original cohort was fairly representative of Sweden as a whole on a number of demographic measures [18]. Moreover, the proportion of temporary employment (8.5%) at age 42 corresponds to the number in Sweden as a whole in the same age group (8.8%) [33]. We were able to adjust for initial health status, measured at age 30, in order to reduce the possibility of reverse causation. We chose to adjust for psychological distress at age 30 but also explored adjustments at age 16 and 21, with comparable inferences. As corresponding measures of self-rated health were collected at age 30 for the first time, we could not explore adjustments for self-rated health at age 16 and 21 but were left to use self-rated health at age 30 as an indicator of previous health. However, the observation that adjustment for psychological distress at age 16 or 21 resulted in less attenuation than did the adjustment at age 30 suggests that health status at age 30 might be an appropriate age for consideration of health selection in this cohort. Further, it is important to consider that health status at age 30 could reflect negative health consequences of peripheral employment before that age. Thus, our findings may underestimate the effects of peripheral employment on health status. Another limitation of this study is that there is a risk of over adjustment when adjusting for socioeconomic position as 'self-employment' is also incorporated into the measure of peripheral employment. Over adjustment would lead to an underestimation of the ORs resulting in a more conservative of the analysis.

The sample size of this study was limited (1005 participants and 433 exposed to peripheral employment), which affects the number of covariates that can be included in the analysis.

All data were based on self-reported data, which might introduce recall bias; this could lead to an underestimation of time in temporary contracts. Comparable measures of self-rated health and psychological distress have displayed validity by predicting future mortality and morbidity [34] even after controlling for known health risks [35]. Moreover, the retrospective measurement of peripheral employment might involve reporting bias. The measurement of temporary employment over an extended period of time is a great asset of this study. However, the sole reliance on self-reported retrospective assessment could introduce recall bias, e.g. that those with health problems over- or under-report the exposure, which could skew the estimates in either direction, or a general under-reporting of the exposure, which would lead to reduced estimates. In addition, difficulties in accurately recalling employment situations over as much as 12 years could also introduce random error of the exposure, which would be expected to reduce the estimated associations. These issues with respect to measurement should be considered when interpreting the results. However, retrospective questions about occupational history have previously been shown to hold good quality in terms of agreement with census data [36]. Furthermore, another limitation regarding the measure of peripheral employment is that a high score could either imply longer duration in peripheral employment positions or shorter duration of a more peripheral employment contract. Also the timing of the exposure is not considered, which would be an important issue to consider in future research. These issues could possibly make the measure less accurate.

### Generalisability

Regarding the generalisability of the results, previous examinations suggest that the original cohort was fairly representative of Sweden as a whole on a number of demographic measures [37]. Moreover, the few participants lost due to attrition, means that participation bias would not be expected to be a major problem. However, the closed nature of the cohort means that the cohort is not as ethnically heterogeneous as the contemporaneous Swedish population. Trade cycles and the regional labour market situation could possibly also influence the impact of temporary employment, e.g. insecure employments might be more stressful in contexts with low availability of jobs. Moreover, all participants were of the same age. This is an important methodological strength as it controls the potentially confounding influence of age by design. However, it is possible that the health impact of temporary employment is moderated by age, e.g. younger people may not have the responsibility of a family to provide for. In summary, caution should be exercised when generalising the results to other ages and ethnic groups, as well as to other labour market contexts. Furthermore, it is possible that the health implications of temporary employment act differently depending on the social context, e.g. that health effects might be more or less prominent depending on structural factors such as national labour market policies, education system and legislation [38].

### Policy implications

Policy changes should aim at reducing health inequalities between those employed in the core and the periphery of the labour market. In this longitudinal study we found that those with experience of long-term peripheral employment have worse health status than those with long-term permanent employment. Although the present study does not focus on specific targets for intervention, our findings suggest that policymakers should aim towards improving the working conditions particularly for those in the peripheral workforce, e.g. health promoting work place measures should also include non-permanent employees. Further, policy measures should help to promote transitions towards the core of the labour market, prevent transitions towards the periphery and help those who potentially are trapped in peripheral employment.

## Conclusions

This study supports the hypothesis of a gradient particularly in psychological distress along with the core-periphery structure, as accumulated over a 12 year period, and that sociodemographics at age 42, previous health and time in unemployment and out of the labour market to a large degree explain these results.

Our results suggest that future research should consider the heterogeneity in temporary employment, including both the variation between different employment contracts and across longer time periods, and the presence of other unfavourable social conditions.

## Competing interests

The authors declare that they have no competing interests.

## Authors' contributions

AH was responsible for the design and data collection. PV and AH contributed to the original idea and conception of the study. AKW was responsible for writing the manuscript and performed all statistical analysis under the supervision of PEG. All authors have contributed to the analysis and interpretations of findings, provided input on manuscript drafts, and have approved the final manuscript.

## Source of support

This work was supported by the Swedish Council for Working Life and Social Research [2006-0950] and the Medical Faculty at Umeå University. Pekka Virtanen was supported by the Academy of Finland: [grant number 132668].

## Pre-publication history

The pre-publication history for this paper can be accessed here:

http://www.biomedcentral.com/1471-2458/11/956/prepub
